# Lectures of M. Agassiz in New York

**Published:** 1847-12

**Authors:** 


*Lectures of M. Agassiz in New York.*—We find in the New York Annalist the following notice of Prof. Agassiz, one of the first of living naturalists. We earnestly hope that we shall have the pleasure of hearing him in Louisville during his stay in our country. We envy our brethren in New York the treat afforded them in these lectures.

This eminent naturalist delivered the introductory lecture of his course upon the general history of the animal kingdom, on Friday evening, October 16, to a very crowded audience. Many were obliged to return, having been unable to procure admission. He alluded to the many different points of view in which natural history may be studied; its importance and interest; exhibited the superiority of man over all other animals, by a reference to the natural construction of his body; proved that he was the last of created animals, and his introduction the object of the successive changes in the geological conditions of the earth and in the animal creation; enumerated the various types which exist in nature; noticed the apparent dissimilarity of similar species; explained the striking and important differences between the types as seen in their nervous systems; and concluded a lecture displaying the deepest learning and the noblest generalizations, and which few men in the country other than himself could have delivered, to the delight and instruction of a most respectable and intelligent audience. He has since delivered to a numerous class three lectures of his regular course, in which he has been engaged in pointing out the peculiarities of structure in the class of the *radiata*, full of the most interesting details and of the most surprising and gratifying proofs of the wisdom and beneficence of the Creator, as displayed in the adaptation of the physical organization of these humble but curious creatures to the wants and purposes of their existence.

M. Agassiz is yet in the prime of life, being but little over forty years of age. He is a little above the common height, of a robust make, has a high, expanded forehead, a pair of well-opened, dark and expressive eyes, and a mouth which is constantly wreathed with a smile of peculiar sweetness and affability. He speaks our language with great accuracy, though with a very decidedly French pronunciation; his voice is loud, clear, and its intonation singularly pleasing. His manner is animated, without being gesticulatory; he aims at no flights of eloquence, but enunciates with the utmost clearness the immense store of knowledge which is so familiar to him, and to him evidently so deeply interesting, and which he contrives easily to make equally so to others. There is about him an earnest honesty of purpose extremely captivating. He is, in the strictest sense of the word, a philosopher. He infers nothing without probability, and asserts nothing without proof. What he knows he knows thoroughly, and tells in the clearest and most intelligible manner. What he does not know, or rather what is not known, he declares with equal candor, and alludes to the results of his own investigations, the fruits of long and untiring industry, with a modesty which is ever the characteristic of true greatness and knowledge. In general society, he has the courtesy of the foreigner, without his usual excess of politeness and compliment. He cheerfully and promptly pours forth the vast amount of knowledge which he has acquired, to all who seek to be informed as to the subjects which have so long and so closely occupied his attention. He is as wholly destitute of hauteur and reserve as he is of garrulity or assumption. He is a truly learned and delightful person, whom none can know without esteem and respect. The influence of his teachings and example cannot fail of being highly beneficial, and has already been felt by many who have attended his lectures—lectures which, we are convinced, will do much to popularize the study of natural history among us. Prof. A. is a man of untiring industry in the pursuit of knowledge. He comes hither to prosecute his favorite study, and, we rejoice to think, will remain in this country for some years. He goes to our markets early every morning in search of new species of his cherished "fishes," which he here meets with abundantly; and can be seen daily, for the greater part of the morning, busily at work in the room kindly allotted to him in the college, dissecting them, and examining other specimens of natural history which are given to him, explaining and demonstrating, to a group of eager listeners, the peculiarities of their organization, or superintending the labors of the three artists who follow in his suite and are constantly engaged in committing to paper the results of his investigations. Delightful task! The sight of Prof. Agassiz in his studio has made us regret that we are not ourselves a naturalist.

We earnestly recommend those of our intelligent readers who have not yet heard Prof. A. to attend at least one of his lectures. It will not, we are sure, be the last, and we will promise them a rich intellectual treat—delight both in the matter of the lectures and in the manner of their delivery—new sources of wonder and admiration for the wisdom of the Creator, new incentives to study, and a new mode of occupying time both pleasantly and profitably, in repeating some at least of his investigations.

